# Genomics and prognosis analysis of epithelial-mesenchymal transition in colorectal cancer patients

**DOI:** 10.1186/s12885-020-07615-5

**Published:** 2020-11-23

**Authors:** Zizhen Zhang, Sheng Zheng, Yifeng Lin, Jiawei Sun, Ning Ding, Jingyu Chen, Jing Zhong, Liuhong Shi, Meng Xue

**Affiliations:** 1 Department of Gastroenterology, the Second Affiliated Hospital of Zhejiang University School of Medicine, Hangzhou, 310020 China; 2 Institution of Gastroenterology, Zhejiang University, Hangzhou, 310000 China

**Keywords:** Colorectal cancer, Epithelial-mesenchymal transition, TCGA, GEO, Signature

## Abstract

**Background:**

The epithelial-mesenchymal transition (EMT) plays a pivotal role in various physiological processes, such as embryonic development, tissue morphogenesis, and wound healing. EMT also plays an important role in cancer invasion, metastasis, and chemoresistance. Additionally, EMT is partially responsible for chemoresistance in colorectal cancer (CRC). The aim of this research is to develop an EMT-based prognostic signature in CRC.

**Methods:**

RNA-seq and microarray data, together with clinical information, were downloaded from The Cancer Genome Atlas (TCGA) and the Gene Expression Omnibus (GEO) databases. A total of 244 differentially expressed EMT-related genes (ERGs) were obtained by comparing the expression between normal and tumor tissues. An EMT-related signature of 11 genes was identified as crucially related to the overall survival (OS) of patients through univariate Cox proportional hazard analysis, least absolute shrinkage and selection operator (LASSO), and Cox regression analysis. Finally, we established a clinical nomogram to predict the survival possibility of CRC patients by integrating clinical characteristics and the EMT-related gene signature.

**Results:**

Two hundred and forty-four differentially expressed ERGs and their enriched pathways were confirmed. Significant enrichment analysis revealed that EMT-related signaling pathway genes were highly related to CRC. Kaplan-Meier analysis revealed that the 11-EMT signature could significantly distinguish high- and low-risk patients in both TCGA and GEO CRC cohorts. In addition, the calibration curves verified fine concordance between the nomogram prediction model and actual observation.

**Conclusion:**

We developed a novel EMT-related gene signature for the prognosis prediction of CRC patients, which could improve the individualized outcome prediction in CRC.

**Supplementary Information:**

The online version contains supplementary material available at 10.1186/s12885-020-07615-5.

## Background

Colorectal cancer (CRC) remains the third leading cause of cancer-related deaths worldwide [1]. Nearly 1.8 million CRC patients are initially diagnosed and 1 million CRC patients die every year [2, 3]. Despite continuous efforts in prevention, screening, and management, the incidence of CRC was still increased by 38% from 2007 to 2017 [2]. In addition, patients with the same clinical and pathologic conditions show contrasting clinical outcomes, even when treated similarly [4]. The diverse prognosis of CRC patients might be due to the inherent genetic heterogeneity.

There is still no definite conclusion about the pathogenesis of CRC. However, a growing number of studies have shown that the epithelial-mesenchymal transition (EMT) plays an important role in invasion, metastasis, and chemoresistance [5–9]. Even though the mechanisms of EMT have been extensively studied in CRC, the prognostic value of ERGs remains limited and inconclusive.

Considering the strong relationship between EMT and tumor pathogenesis, the aim of this study is to identify ERGs for cancer diagnosis, management, and prognosis. We initially screened differentially expressed ERGs between tumorous and nontumorous tissues, and then used Cox proportional hazard regression analysis to screen prognosis-related genes from 244 EMT-associated genes in a CRC cohort of The Cancer Genome Atlas (TCGA). The resulting genes were applied to the least absolute shrinkage and selection operator (LASSO) to establish an optimal risk model, followed by validation in an independent Gene Expression Omnibus (GEO) CRC population. The results showed that CRC patients with high EMT risk scores were obviously associated with shorter overall survival (OS) than that of patients with low risk scores. The difference in the key signaling pathways between high and low risk groups were explored using gene set enrichment analysis (GSEA). Taken together, our research constructs a nomogram to predict individuals’ survival probability by integrating clinical characteristics and the prognostic gene signature.

## Methods

### Data processing

ERGs were downloaded from the Epithelial-Mesenchymal Transition Gene Database (http://dbemt.bioinfo-minzhao.org/download.cgi) and the Molecular Signatures Database v7.1 (http://www.broadinstitute.org/gsea/msigdb/index.jsp). We listed the all EMT-related genes in Table S1. We downloaded the RNA-seq data and clinical information for CRC from the TCGA database (https://portal.gdc.cancer.gov/). The GSE17536 dataset was obtained from the GEO (https://www.ncbi.nlm.nih.gov/geo/) for the validation studies.

### Differentially expressed ERGs and enrichment analysis

The differentially expressed ERGs in the mRNA expression data of the CRC cohort were identified by the limma package in R software (version 3.6.1) (adjusted *P* < 0.05, |logFC| > 1) [10]. Volcano plots and heat maps were visualized with the ggrepel, ggplot, and pheatmap packages in R software. Entrez gene annotations were referred to as “org. Hs.eg.db”. The functional annotation of Gene Ontology (GO), including biological process (BP), cellular component (CC), and molecular function (MF), was performed in the R “clusterProfiler” [11]. The GO cluster was plotted with the R “GOplot” package [12]. Kyoto Encyclopedia of Genes and Genomes (KEGG) pathway enrichment analysis was performed using the open access WebGestalt tool (http://www.webgestalt.org) [13, 14]. The results with a false discovery rate (FDR) ≤ 0.05 were included. The enriched pathways and processes were visualized in the volcano plot, where the x- and y-axis showed the enrichment ratio and the log of the FDR for all categories in the database [13, 14]. In addition, we used GSEA to uncover the different signaling pathways between high- and low-risk subgroups (http://software.broadinstitute.org/gsea/). The number of random sample permutations was set at 1000, and the significance threshold was set at *p* < 0.05.

### Functional enrichment of protein-protein interaction network of ERGs

The STRING database was applied to construct potential protein-protein interactions (PPI) among the ERGs [15]. PPI pairs with a combined score > 0.4 were extracted. The connectivity degree of each node in the network was calculated. Then, the PPI network was constructed based on these protein pairs using Cytoscape software [16]. Moreover, the genes of the prognostic model were used to identify interactions between proteins through GeneMANIA [17, 18].

### Construction and validation of EMT-related gene signature

Univariate Cox regression analysis was used to identify genes clearly related to OS with *p*-values < 0.01. Then, the significant prognostic genes were filtered in LASSO-penalized Cox regression analysis. A λ value of 0.023 with log (λ) = − 3.78 was selected by 10-fold cross-validation via minimum criteria. Only genes with nonzero coefficients in the LASSO regression model were chosen to further calculate the risk score [19]. The formula used to calculate the degree of crystallization is presented in Eq. (1). In Eq. (1), n denotes the number of prognostic genes, Gi represents the expression value of the ith genes, and weight i represents the coefficient of each gene. The same formula was used to calculate risk scores in GEO datasets, as in the TCGA datasets. We used Kaplan–Meier survival curves and the log-rank method to estimate the prognostic significance. A *p*-value < 0.05 was considered statistically significant. Receiver operating characteristic (ROC) analyses were performed in R “survival ROC” to validate gene signatures in TCGA and GSE17536 datasets.

1 $$ \mathrm{Risk}\ \mathrm{score}={\sum}_{i=1}^n{G}_i\ast {\mathrm{weight}}_i $$

### Development of nomogram

Age, gender, stage, and risk score were used to construct a nomogram, using the survival and rms package for R. Moreover, calibration curves were plotted to assess the concordance between actual and predicted survival. Furthermore, decision curve analysis (DCA) was used to measure whether our established nomogram was suitable for clinical utility. The x-axis represents the percentage of threshold probability, and the y-axis represents the net benefit.

### Statistical analysis

All statistical analyses were carried out with R (version 3.6.0). Kaplan-Meier survival analysis was used to estimate the survival differences between the high- and low-risk groups in the datasets. Univariate and multivariate Cox proportional hazard regression analyses were performed to determine prognostic values for risk scores, as well as various clinical features.

To validate the effect of the risk assessment model, we used the ROC curve for verification. The calibration curves and DCA were applied to determine the predictive accuracy of the prognostic models.

## Results

### Identification of ERGs in CRC

**Fig. 1:**
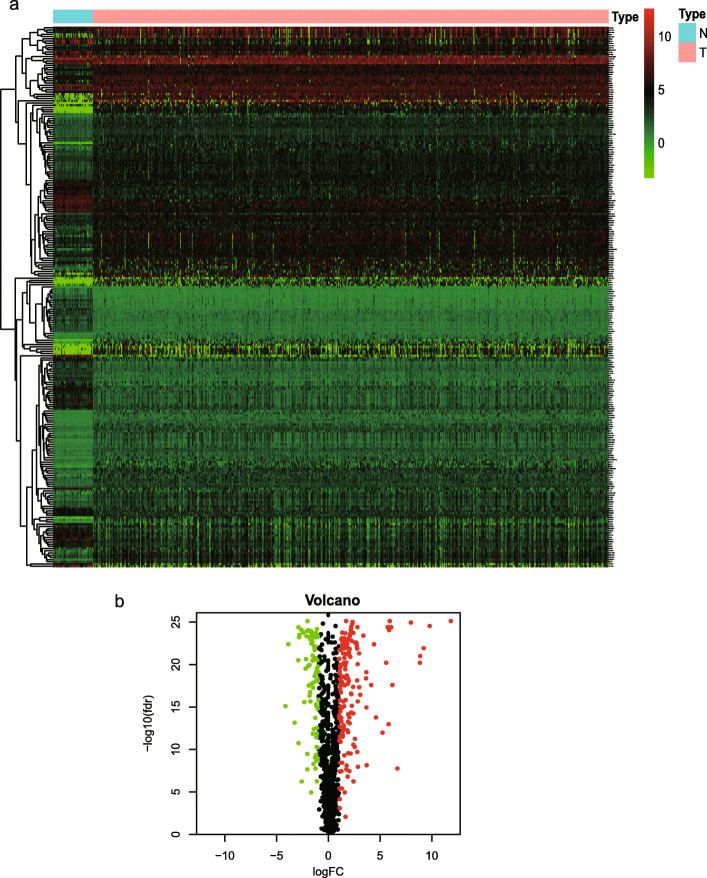
Differentially expressed ERGs between colorectal cancer (CRC) and normal colorectal tissues. **a** Heatmap for differentially expressed ERGs. **b** Volcano plot of differentially expressed mRNAs between CRC and normal tissues. The heatmap was generated using version 3.6.1 of R software

The flowchart of this study is shown in Fig. S1. All the mRNA expression profiles and clinical follow-up data of 568 cancer samples and 44 normal samples from TCGA dataset were downloaded, containing 1269 ERGs. Among these, 1121 genes of intersect expression in TCGA dataset and GSE17536 were selected, and then normal samples and CRC samples were compared through the limma package in R software (adjusted *P* < 0.05, |log2-fold change| > 1). There were 159 genes significantly upregulated and 85 genes significantly downregulated in CRC. Figure 1a revealed a heatmap of differentially expressed mRNAs between groups. The volcano map was shown in Fig. 1b**.**

### Biological functions and significant pathway analysis

**Fig. 2:**
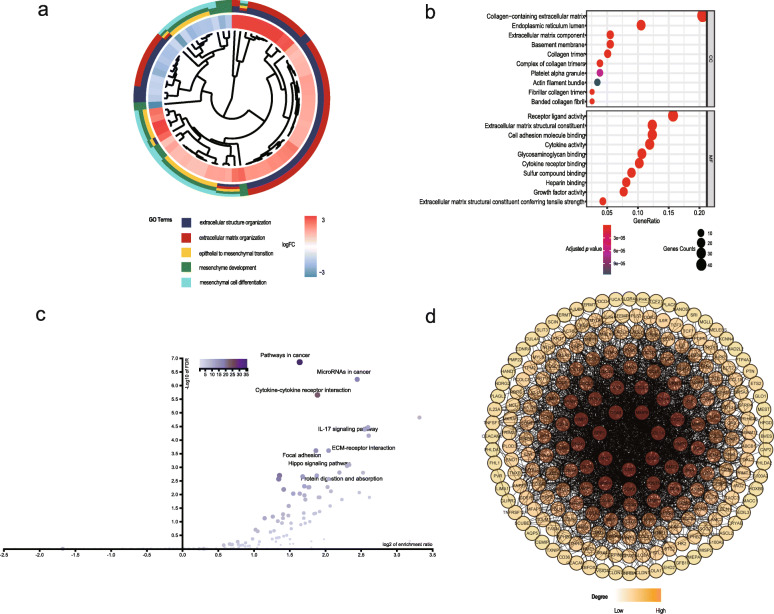
GO, KEGG analysis, and protein-protein interaction (PPI) network of ERGs. **a** GO cluster. The inner dendrogram indicates the hierarchical clustering of the gene expression profiles; the outer circle represents the log2FC of each ERG, with the color corresponding to the gene level; and the outermost circle represents the GO BP terms assigned to the gene. **b** The 10 most significantly enriched CC and MF terms. **c** Volcano plot of EMT gene-associated pathways. **d** PPI network of all differentially expressed ERGs

The functions and significant pathways of these 244 differentially expressed ERGs were identified by GO enrichment and KEGG pathway analyses. GO enrichment terms are shown in Fig. 2a and b**.** The analysis showed a significant enrichment of processes related to the growth of CRC and the EMT process. KEGG pathway enrichment of these genes was mainly associated with focal adhesion, the Hippo signaling pathway, and IL-17 signaling pathways (Fig. 2c). These genes were linked and formed a tight PPI network, as indicated in Fig. 2d.

### Identification of survival-related differentially expressed ERGs

The correlations between the differentially expressed ERGs and clinical data were analyzed using univariate Cox regression (*p* < 0.01 is considered significant). Sixteen genes were screened with prognostic value in CRC.

### Establishment and validation of the prognostic model

**Fig. 3:**
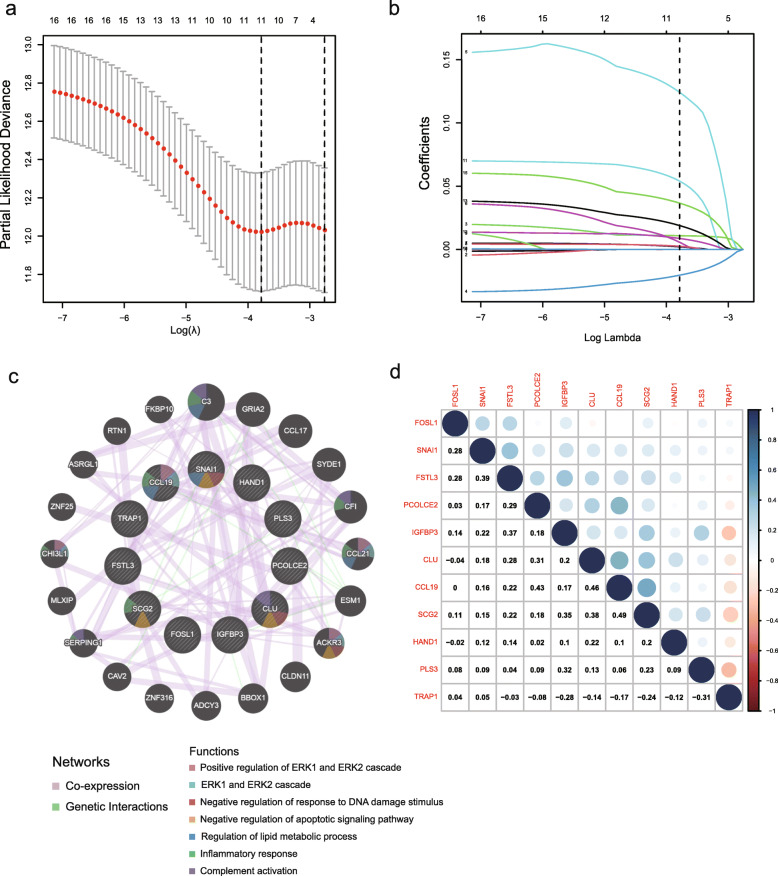
Establishment of prognostic gene signature by LASSO regression analysis. **a** Solid vertical lines represent partial likelihood deviance ± standard error (SE). The dotted vertical lines are drawn at the optimal values by minimum criteria and 1-SE criteria. Herein, a value λ = 0.023 with log (λ) = − 3.78 was chosen by 10-fold cross-validation via minimum criteria. **b** Selection of the optimal parameter (lambda) in the LASSO model for training cohort. **c** Gene-gene interaction network among selected genes by LASSO regression analysis in the GeneMANIA dataset. **d** Spearman’s correlation analysis of the selected genes by LASSO regression analysis

The 16 EMT-related genes were filtered into LASSO-penalized Cox regression analysis (Fig. 3a and b). After 1000 resamples, an 11-gene prognostic model, including follistatin-like 3 (FSTL3), TNF receptor-associated protein 1 (TRAP1), procollagen C-endopeptidase enhancer 2 (PCOLCE2), secretogranin II (SCG2), clusterin (CLU), C-C motif chemokine ligand 19 (CCL19), heart and neural crest derivatives expressed 1 (HAND1), FOS-like 1 (FOSL1), AP-1 transcription factor subunit (FOSL1), plastin 3 (PLS3), insulin-like growth factor binding protein 3 (IGFBP3), and snail family transcriptional repressor 1 (SNAI1)—was constructed. We used GeneMANIA to analyze the relationships between the 11 genes (Fig. 3c). A strong correlation was noticed in the genetic interaction between CCL19 and SCG2, as well as between CCL19 and PCOLCE2. Furthermore, a majority of the 11 genes were correlated with each other (Fig. 3d). The risk score = (0.011 × FSTL3) + (− 0.02 × TRAP1) + (0.124 × PCOLCE2) + (0.0057 × SCG2) + (0.00212 × CLU) + (0.00257 × CCL19) + (0.054 × HAND1) + (0.00874 × FOSL1) + (0.0189 × PLS3) + (0.0000763 × IGFBP3) + (0.0366 × SNAI1). The samples were classified into high-risk and low-risk groups according to the median risk score.

**Fig. 4:**
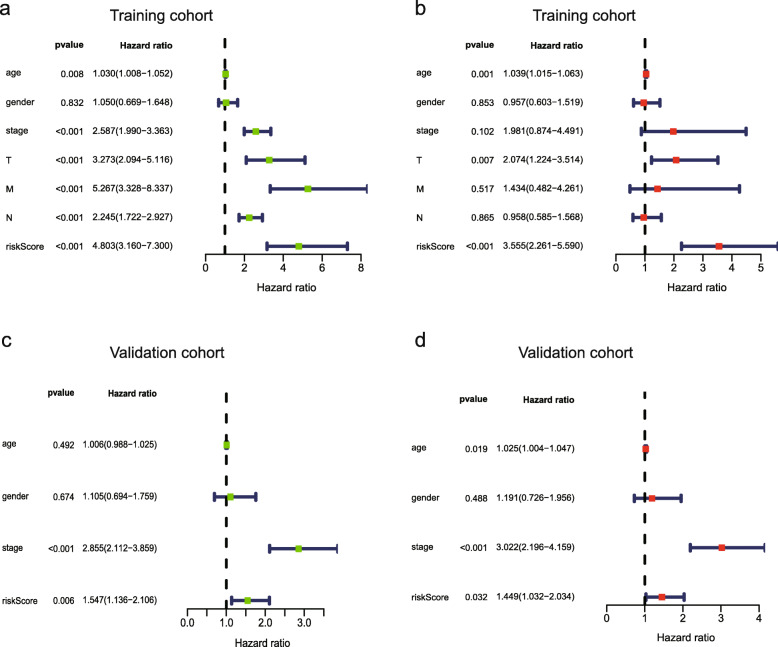
Forrest plot of the univariate and multivariate Cox regression analysis. **a**, **c** Univariate Cox proportion hazard regression for OS of CRC in training and validation cohorts. **b**, **d** Multivariable Cox proportion hazard regression for OS of CRC in training and validation cohorts

**Fig. 5:**
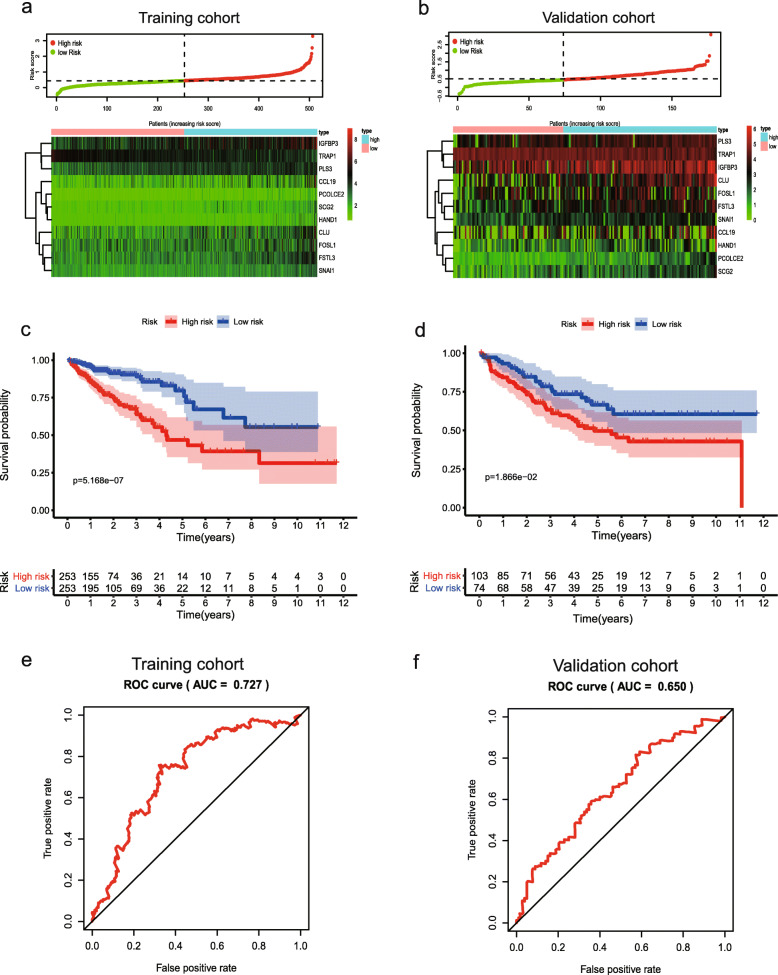
Development of risk score based on the 11-EMT-related gene signature of patients with CRC in TCGA and GEO. **a**, **b** The hierarchical clustering analysis of eleven genes with the increase of the risk score. **c**, **d** Kaplan–Meier analysis of the prognostic model in TCGA or GEO datasets. **e**, **f** Time-dependent ROC analysis showing the optimal AUC of the gene signature in the two cohorts

**Fig. 6:**
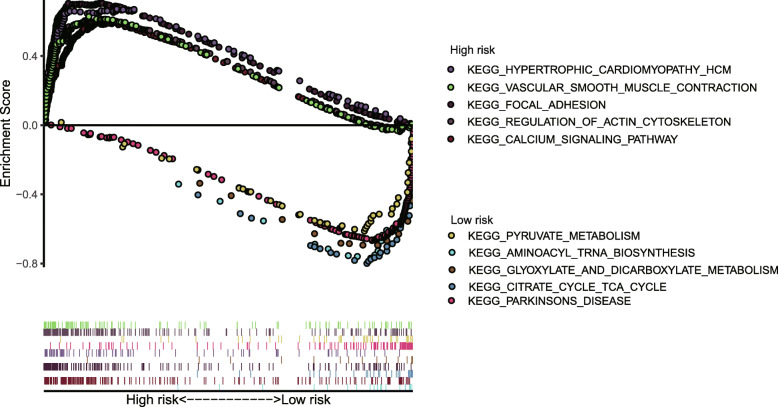
Ten representative enriched KEGG pathways by GESA. Each group contains five KEGG pathways. GESA, gene set enrichment analysis; KEGG, Kyoto Encyclopedia of Genes and Genomes

Univariate Cox analysis and multiple Cox regression analysis verified whether the model could be independent progress factors (Fig. 4). The results demonstrated that the lower survival outcome of CRC patients was related to higher risk score (Fig. 5a and b). The Kaplan–Meier analysis displayed a significant difference in the outcome of the patients between the high-risk group and the low-risk group (log-rank test *p* < 0.001; Fig. 5c and d). The area under the ROC curve (AUC) for the model was 0.727 and 0.65 in TCGA and GEO datasets, respectively (Fig. 5e and f). Meanwhile, we also evaluated angiogenesis related genes and metabolism-related genes as genetic indicators for survival prediction. The corresponding AUC values were 0.538 and 0.584 respectively, which are not yet ideal (Fig. S2). In addition, we used GSEA to uncover the different signaling pathways between high- and low-risk subgroups. The representative pathways were showed in Fig. 6**.**

### Construction of a nomogram

**Fig. 7:**
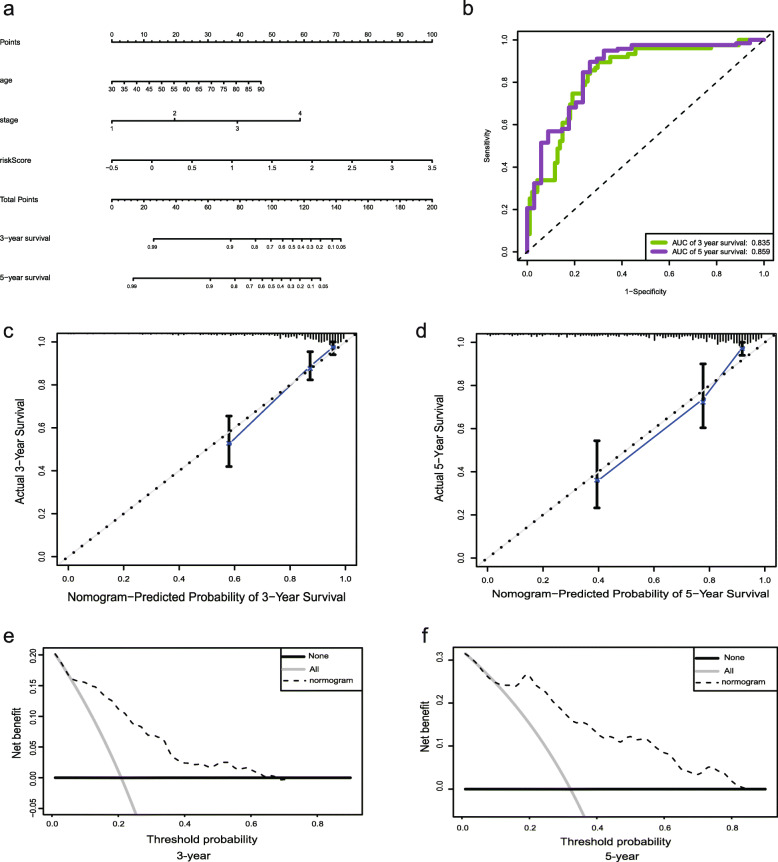
Construction of a nomogram based on the 11-EMT-related gene signature. **a** A nomogram based on the signature and clinical information. **b** Time-dependent receiver operating characteristic (ROC) curve for predicting overall survival (OS) of the nomogram. **c**, **d** Calibration plot evaluating the predictive accuracy of the nomogram [at 3-year survival (**c**) at 5-year survival (**d**)]. (e, f) Decision curve analysis evaluating the clinical utility of the nomogram [at 3-year survival (**e**) at 5-year survival (**f**)]

According to the Cox regression combined with the significant clinical parameters, the nomogram contains three prognostic parameters: age, stage, and risk score (Fig. 7a). Every patient receives one point for each prognostic parameter, and higher total points indicates a worse outcome. Moreover, the ROC curves of 3- and 5-year OS shows that our model has a good predictive ability (Fig. 7b). The calibration plots indicated that in comparison with an ideal model, the nomogram had a similar performance (Fig. 7c). The results of DCA also demonstrated that our nomogram has high potential for clinical utility (Fig. 7d).

## Discussion

Nowadays, CRC remains a major threat to human health, but the mechanisms underlying its pathogenesis are still unclear. However, it is significant for researchers to explore new diagnostic and therapeutic strategies. On the other hand, an increasing number of studies have widely proved that EMT plays an important role in the development and progression of CRC [20]. Recently, mRNA gene signatures based on certain characteristics, such as metabolism [21] and cell cycle [22], have become research hotspots.

In this study, we collected the transcriptome data along with their corresponding clinical information from TCGA and GEO databases. Among these, we obtained the differentially expressed ERGs between CRC samples and nontumorous samples. Further analysis was performed to identify the oncogenes. Finally, a prognostic model for CRC patients was constructed. Interestingly, major differentially expressed ERGs were enriched in several cancer-related pathways-the Hippo signaling pathway, ERK1 and ERK2 cascade, negative regulation of response to DNA damage stimulus, and so on. Notably, it has been reported that the IL-17 pathway participated in autoimmune pathology or hypersensitivity, host defense, and tissue repair [23]. Consistent with previous findings [24, 25], we predicted that the IL-17 pathway might be involved in the EMT process through KEGG enrichment. Interestingly, IL-17 upregulated PD-L1 protein expression in HCT116 and LNCaP cells, as reported in previous literature [26]. Therefore, targeting this pathway could not only inhibit the tumor metastasis, but also enhance the killing effect of immune cells on tumors.

Eleven genes were used to establish the model equations for risk assessment. Among them, three candidate genes (FOSL1, PLS3, SNAI1) were reported to promote CRC cell migration and invasion. FOSL1 plays a central role in EMT and is highly expressed in solid cancers, especially in metastatic CRC. In vitro studies showed that blocking the expression of FOSL1 could diminish the migration of tumor cells [27]. Mimori et al. confirmed that PLS3 induced EMT via transforming growth factor (TGF)- β signaling, followed by the acquisition of invasive ability in CRC cells. Furthermore, overexpression of PLS3 in CRC cells significantly increased the expression levels of EMT-related transcription factors (TWIST, SNAI1, SLUG, SMAD4, and ZEB1), EMT markers (vimentin, FN1, and N-cadherin), and TGF-β, enhancing the invasiveness of CRC cells [28]. In addition, previous studies have demonstrated that high expression of PLS3 in peripheral blood was independently associated with poor prognosis and recurrence [29]. Wang et al. identified that SNAI1 was not detected in normal colorectal epithelia, whereas SNAI1 was upregulated in tumor tissues from lymph node (LN) + patients [30]. Similar studies have found that SNAI1 was upregulated in CRC, which might have potential in the control of metastasis and possibly serve as a target for chemopreventive agents [31]. Data from Gene Expression Profiling Interactive Analysis (GEPIA) revealed that a high expression of TRAP1 was correlated with a good prognosis in CRC. However, researchers have already observed that TRAP1 was significantly upregulated in CRC patients with lymph node metastasis compared to those without LM metastasis [32]. Using RT-qPCR detection of CRC in different tumor stages, Scorilas et al. found that the CLU mRNA expression levels were significantly enhanced as CRC tumors progressed from tumor node metastasis (TNM) stage I to IV [33]. Further in vivo and in vitro experiments focusing on TRAP1 and CLU are still needed to explore their roles in CRC.

Of note, contrary to our research, Zhou et al. confirmed that decreased expression of IGFBP3 promoted tumor metastasis in CRC [34]. Another study indicated that silencing IGFBP3 in two human CRC cell lines, SW480 and Caco2, could reduce the proliferation, colony formation, and migration. They found that the expression levels of IGFBP3 simultaneously increased with the growth and advanced stage of CRC [35]. Our studies, however, showed that increased IGFBP3 expression was associated with a poor prognosis in CRC patients. Considering the inconsistent results, further experiments are still required. As a potential immune stimulator, CCL19 has been observed to be increased in lung cancer, and an association between CCL19 expression and high TNM staging and vascular invasion was identified [36]. CCL19 enhances parenchymal central nervous system (CNS) retention of lymphoma cells (LCs), thereby promoting central nervous system lymphoma (CNSL) formation [37]. Xu et al. identified that CCL19 suppressed angiogenesis in CRC via promoting miR-206 [38]. However, further study will be required to uncover and understand its mechanisms in the metastasis of CRC.

FSTL3 was upregulated by the lncRNA DSCAM-AS1/miR-122-5p axis and could promote proliferation and migration of non-small cell lung cancer cells [39]. Moreover, FSTL3 served as a surrogate marker in breast cancer and was the only variable that could distinguish a benign breast mass from a malignant one [40]. One report indicated that astrocytic HAND1 was found to be unique in metastatic gastrointestinal stromal tumor (GIST) and might work as a transcriptional amplifier of the oncogenic GIST program [41]. There are few studies on these two genes in CRC. Further research on these genes is required. It should be noted that SCG2 and PCOLCE2 have been predicted to be associated with the prognosis of CRC, but in-depth investigation on these two genes in CRC is rarely reported [42, 43]. It is necessary to explore their roles in tumors, especially in CRC.

So far, most of the cancer-related genes identified through bioinformatics methods were analyzed individually, which could not reflect the carcinogenesis process comprehensively. However, we generated a multigene signature predicting the prognosis of individual CRC patients, focusing on the ERG sets. Nevertheless, this research also has some imperfections. First, we examined data from public databases, so the quality could hardly be guaranteed. Second, the study could be more valuable if further experiments in CRC cells and animal models are performed on these genes. Finally, most of the data we studied were obtained from the United States or Europe. Due to the limited origin of the data, they might not be able to reflect all persons worldwide. Therefore, future research is needed to validate our findings.

## Conclusions

In summary, by using 2 datasets, our research established and validated a novel EMT-related gene signature for the prognosis prediction of CRC patients, where higher risk scores indicate poorer prognosis. Further elucidating the underlying mechanisms of these genes will provide theoretical guidance for basic research and better evidence for future clinical decision-making.

## Supplementary Information

**Additional file 1: Figure S1.** Flowchart of the present study.

**Additional file 2: Figure S2.** ROC curve analysis of other genetic indicators. (a) ROC curve based on angiogenesis related genes genes. (b) ROC curve based on metabolism-related genes.

**Additional file 3: Table S1.** All EMT-related genes from the database.

## Data Availability

The datasets used and/or analyzed during the current study are available from The Cancer Genome Atlas (TCGA) repository: https://portal.gdc.cancer.gov/repository?facetTab=cases; Gene Expression Omnibus (GEO) repository: https://www.ncbi.nlm.nih.gov/geo/query/acc.cgi?acc=GSE17536; the Epithelial-Mesenchymal Transition Gene Database: http://dbemt.bioinfo-minzhao.org/download.cgi; and the Molecular Signatures Database v7.1: https://www.gsea-msigdb.org/gsea/msigdb/cards/HALLMARK_EPITHELIAL_MESENCHYMAL_TRANSITION.html.

## Abbreviations

EMT: Epithelial mesenchymal transition
CRC: Colorectal cancer
TCGA: The Cancer Genome Atlas
GEO: Gene Expression Omnibus
ERGs: EMT-related genes
OS: Overall survival
LASSO: Least absolute shrinkage and selection operator
GSEA: Gene set enrichment analysis
GO: Gene Ontology
BP: Biological process
CC: Cellular component
MF: Molecular function
KEGG: Kyoto Encyclopedia of Genes and Genomes
FDR: False discovery rate
PPI: Protein-protein interaction
ROC: Receiver operating characteristic
DCA: decision curve analysis

## References

[1] Siegel RL, Miller KD, Jemal A (2020). Cancer statistics, 2020. CA Cancer J Clin.

[2] Ferlay J, Colombet M, Soerjomataram I, Mathers C, Parkin DM, Piñeros M (2019). Estimating the global cancer incidence and mortality in 2018: GLOBOCAN sources and methods. Int J Cancer.

[3] Siskova A, Cervena K, Kral J, Hucl T, Vodicka P, Vymetalkova V (2020). Colorectal adenomas-genetics and searching for new molecular screening biomarkers. Int J Mol Sci.

[4] Inadomi JM (2017). Screening for colorectal Neoplasia. N Engl J Med.

[5] Fischer KR, Durrans A, Lee S, Sheng J, Li F, Wong ST (2015). Epithelial-to-mesenchymal transition is not required for lung metastasis but contributes to chemoresistance. Nature..

[6] Boesch M, Spizzo G, Seeber A (2018). Concise review: aggressive colorectal Cancer: role of epithelial cell adhesion molecule in Cancer stem cells and epithelial-to-Mesenchymal transition. Stem Cells Transl Med.

[7] Battaglin F, Puccini A, Intini R, Schirripa M, Ferro A, Bergamo F (2018). The role of tumor angiogenesis as a therapeutic target in colorectal cancer. Expert Rev Anticancer Ther.

[8] Li N, Babaei-Jadidi R, Lorenzi F, Spencer-Dene B, Clarke P, Domingo E (2019). An FBXW7-ZEB2 axis links EMT and tumour microenvironment to promote colorectal cancer stem cells and chemoresistance. Oncogenesis..

[9] Gavert N, Ben-Ze'ev A (2008). Epithelial-mesenchymal transition and the invasive potential of tumors. Trends Mol Med.

[10] Ritchie ME, Phipson B, Wu D, Hu Y, Law CW, Shi W (2015). limma powers differential expression analyses for RNA-sequencing and microarray studies. Nucleic Acids Res.

[11] Yu G, Wang LG, Han Y, He QY (2012). clusterProfiler: an R package for comparing biological themes among gene clusters. Omics..

[12] Walter W, Sánchez-Cabo F, Ricote M (2015). GOplot: an R package for visually combining expression data with functional analysis. Bioinformatics..

[13] Zhang B, Kirov S, Snoddy J (2005). WebGestalt: an integrated system for exploring gene sets in various biological contexts. Nucleic Acids Res.

[14] Liao Y, Wang J, Jaehnig EJ, Shi Z, Zhang B (2019). WebGestalt 2019: gene set analysis toolkit with revamped UIs and APIs. Nucleic Acids Res.

[15] Szklarczyk D, Morris JH, Cook H, Kuhn M, Wyder S, Simonovic M (2017). The STRING database in 2017: quality-controlled protein-protein association networks, made broadly accessible. Nucleic Acids Res.

[16] Shannon P, Markiel A, Ozier O, Baliga NS, Wang JT, Ramage D (2003). Cytoscape: a software environment for integrated models of biomolecular interaction networks. Genome Res.

[17] Mostafavi S, Ray D, Warde-Farley D, Grouios C, Morris Q (2008). GeneMANIA: a real-time multiple association network integration algorithm for predicting gene function. Genome Biol.

[18] Warde-Farley D, Donaldson SL, Comes O, Zuberi K, Badrawi R, Chao P (2010). The GeneMANIA prediction server: biological network integration for gene prioritization and predicting gene function. Nucleic Acids Res.

[19] Kidd AC, McGettrick M, Tsim S, Halligan DL, Bylesjo M, Blyth KG (2018). Survival prediction in mesothelioma using a scalable Lasso regression model: instructions for use and initial performance using clinical predictors. BMJ Open Respir Res.

[20] Brabletz T, Hlubek F, Spaderna S, Schmalhofer O, Hiendlmeyer E, Jung A (2005). Invasion and metastasis in colorectal cancer: epithelial-mesenchymal transition, mesenchymal-epithelial transition, stem cells and beta-catenin. Cells Tissues Organs.

[21] Zhang ZY, Yao QZ, Liu HY, Guo QN, Qiu PJ, Chen JP (2020). Metabolic reprogramming-associated genes predict overall survival for rectal cancer. J Cell Mol Med.

[22] Zhao L, Jiang L, He L, Wei Q, Bi J, Wang Y (2019). Identification of a novel cell cycle-related gene signature predicting survival in patients with gastric cancer. J Cell Physiol.

[23] Li X, Bechara R, Zhao J, McGeachy MJ, Gaffen SL (2019). IL-17 receptor-based signaling and implications for disease. Nat Immunol.

[24] Chen Y, Yang Z, Wu D, Min Z, Quan Y (2019). Upregulation of interleukin-17F in colorectal cancer promotes tumor invasion by inducing epithelial-mesenchymal transition. Oncol Rep.

[25] Zhang Q, Liu S, Parajuli KR, Zhang W, Zhang K, Mo Z (2017). Interleukin-17 promotes prostate cancer via MMP7-induced epithelial-to-mesenchymal transition. Oncogene..

[26] Wang X, Yang L, Huang F, Zhang Q, Liu S, Ma L (2017). Inflammatory cytokines IL-17 and TNF-α up-regulate PD-L1 expression in human prostate and colon cancer cells. Immunol Lett.

[27] Diesch J, Sanij E, Gilan O, Love C, Tran H, Fleming NI (2014). Widespread FRA1-dependent control of mesenchymal transdifferentiation programs in colorectal cancer cells. PLoS One.

[28] Sugimachi K, Yokobori T, Iinuma H, Ueda M, Ueo H, Shinden Y (2014). Aberrant expression of plastin-3 via copy number gain induces the epithelial-mesenchymal transition in circulating colorectal cancer cells. Ann Surg Oncol.

[29] Yokobori T, Iinuma H, Shimamura T, Imoto S, Sugimachi K, Ishii H (2013). Plastin3 is a novel marker for circulating tumor cells undergoing the epithelial-mesenchymal transition and is associated with colorectal cancer prognosis. Cancer Res.

[30] Fan XJ, Wan XB, Yang ZL, Fu XH, Huang Y, Chen DK (2013). Snail promotes lymph node metastasis and Twist enhances tumor deposit formation through epithelial-mesenchymal transition in colorectal cancer. Hum Pathol.

[31] Roy HK, Smyrk TC, Koetsier J, Victor TA, Wali RK (2005). The transcriptional repressor SNAIL is overexpressed in human colon cancer. Dig Dis Sci.

[32] Gao JY, Song BR, Peng JJ, Lu YM (2012). Correlation between mitochondrial TRAP-1 expression and lymph node metastasis in colorectal cancer. World J Gastroenterol.

[33] Artemaki PI, Sklirou AD, Kontos CK, Liosi AA, Gianniou DD, Papadopoulos IN (2020). High clusterin (CLU) mRNA expression levels in tumors of colorectal cancer patients predict a poor prognostic outcome. Clin Biochem.

[34] Zhou N, Sun Z, Li N, Ge Y, Zhou J, Han Q (2018). miR-197 promotes the invasion and migration of colorectal cancer by targeting insulin-like growth factor-binding protein 3. Oncol Rep.

[35] Georges RB, Adwan H, Hamdi H, Hielscher T, Linnemann U, Berger MR (2011). The insulin-like growth factor binding proteins 3 and 7 are associated with colorectal cancer and liver metastasis. Cancer Biol Ther.

[36] Liu Y, Wu BQ, Geng H, Xu ML, Zhong HH (2015). Association of chemokine and chemokine receptor expression with the invasion and metastasis of lung carcinoma. Oncol Lett.

[37] O'Connor T, Zhou X, Kosla J, Adili A, Garcia Beccaria M, Kotsiliti E (2019). Age-Related Gliosis Promotes Central Nervous System Lymphoma through CCL19-Mediated Tumor Cell Retention. Cancer Cell.

[38] Xu Z, Zhu C, Chen C, Zong Y, Feng H, Liu D (2018). CCL19 suppresses angiogenesis through promoting miR-206 and inhibiting met/ERK/Elk-1/HIF-1α/VEGF-A pathway in colorectal cancer. Cell Death Dis.

[39] Gao L, Chen X, Wang Y, Zhang J (2020). Up-regulation of FSTL3, regulated by lncRNA DSCAM-AS1/miR-122-5p Axis, promotes proliferation and migration of non-small cell lung Cancer cells. Onco Targets Ther.

[40] Panagiotou G, Papakonstantinou E, Vagionas A, Polyzos SA, Mantzoros CS (2019). Serum levels of Activins, Follistatins, and growth factors in neoplasms of the breast: a case-control study. J Clin Endocrinol Metab.

[41] Hemming ML, Lawlor MA, Zeid R, Lesluyes T, Fletcher JA, Raut CP (2018). Gastrointestinal stromal tumor enhancers support a transcription factor network predictive of clinical outcome. Proc Natl Acad Sci U S A.

[42] Sun G, Li Y, Peng Y, Lu D, Zhang F, Cui X (2019). Identification of a five-gene signature with prognostic value in colorectal cancer. J Cell Physiol.

[43] Chen L, Lu D, Sun K, Xu Y, Hu P, Li X (2019). Identification of biomarkers associated with diagnosis and prognosis of colorectal cancer patients based on integrated bioinformatics analysis. Gene..

