# Supplementary figures and images for: Genomics and prognosis analysis of epithelial-mesenchymal transition in colorectal cancer patients
Source: BMC Cancer. 2020 Nov 23;20:1135. doi: 10.1186/s12885-020-07615-5 (PMC7686680; doi:10.1186/s12885-020-07615-5)

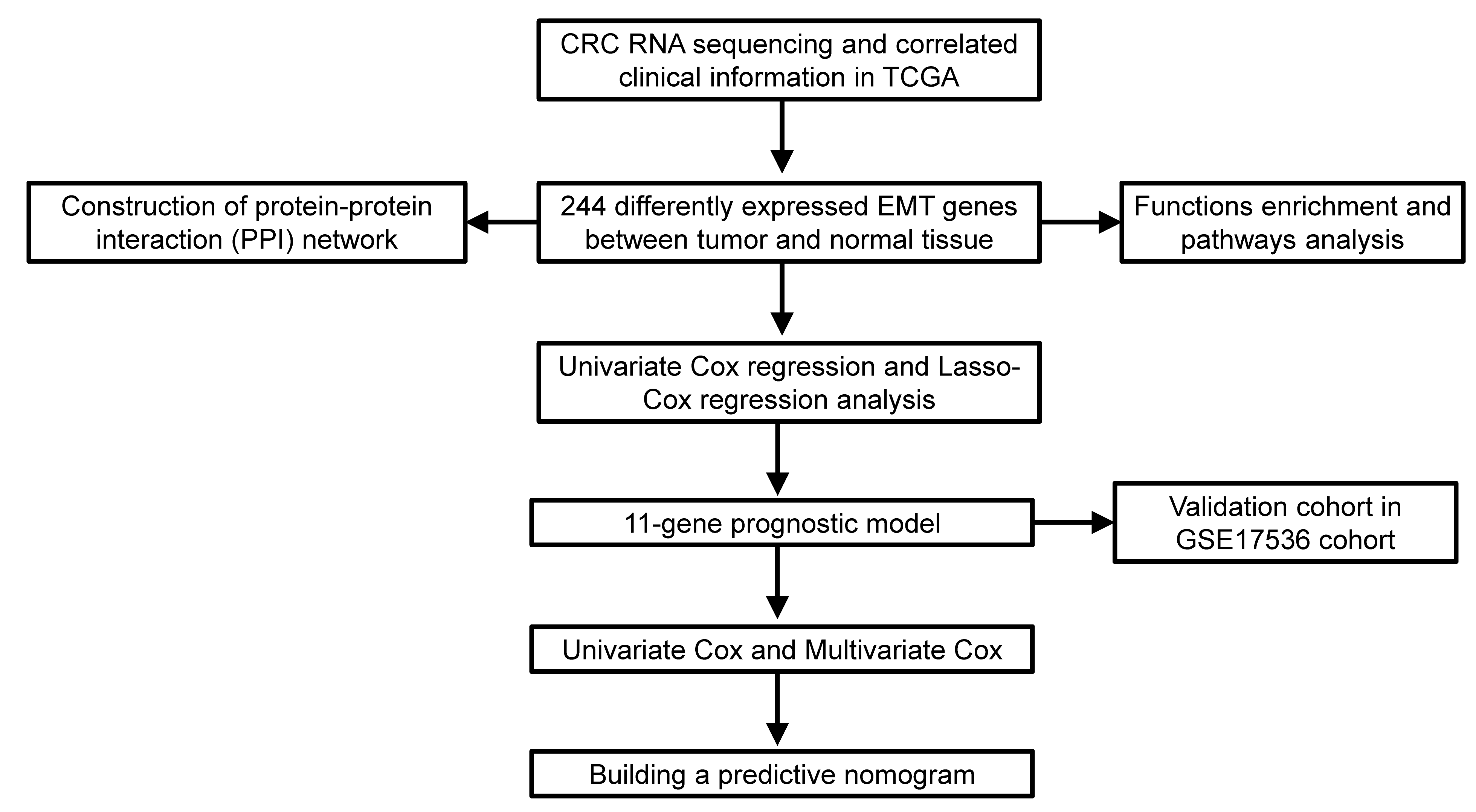

Supplement: Supplementary file 1 — Additional file 1: Figure S1. Flowchart of the present study. [file 12885_2020_7615_MOESM1_ESM.tif]

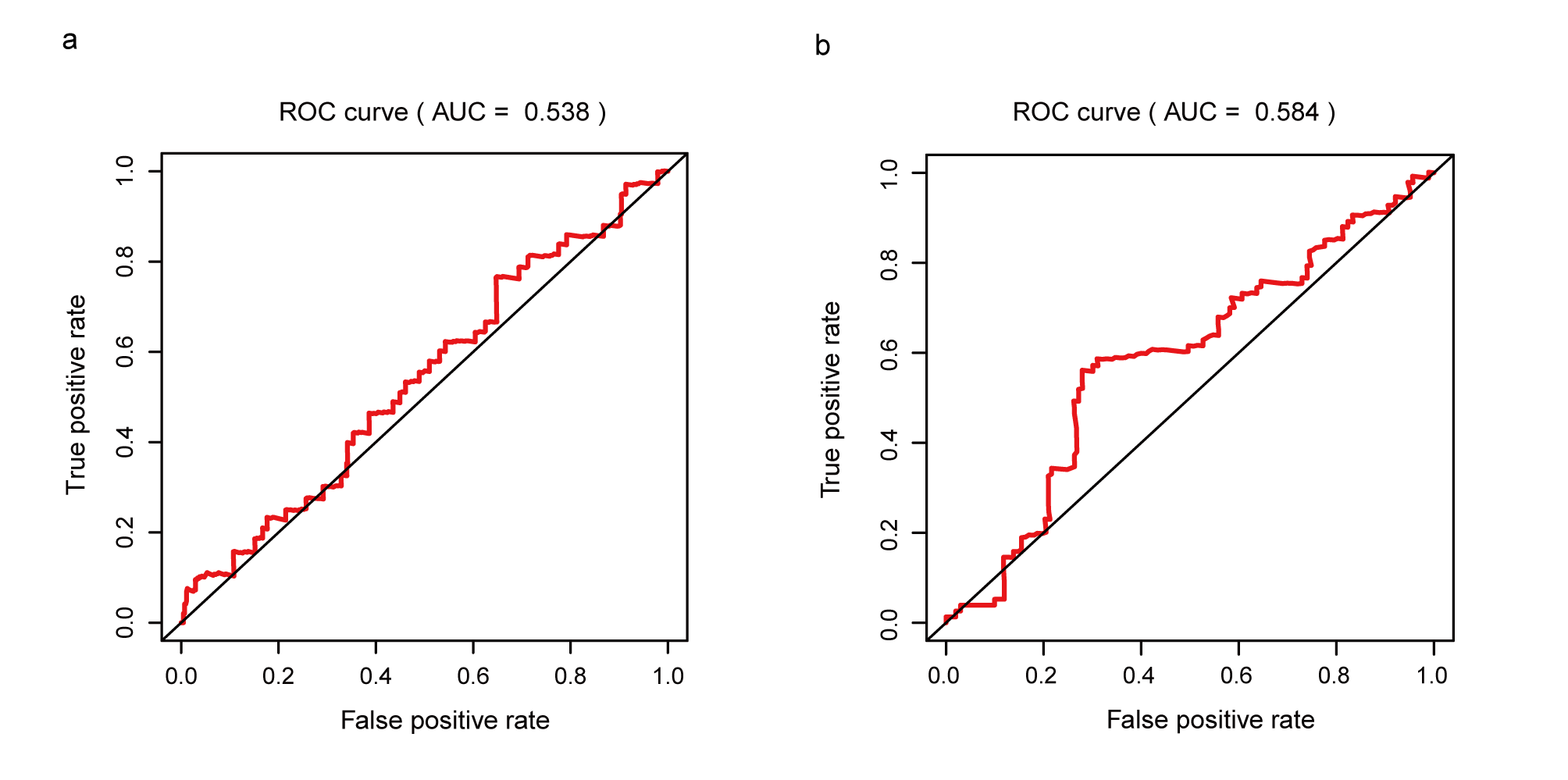

Supplement: Supplementary file 2 — Additional file 2: Figure S2. ROC curve analysis of other genetic indicators. (a) ROC curve based on angiogenesis related genes genes. (b) ROC curve based on metabolism-related genes. [file 12885_2020_7615_MOESM2_ESM.tif]
